# Interhemispheric Functional Hypoconnectivity Is an Early Marker of Cortical Epileptogenesis

**DOI:** 10.3390/biomedicines14030549

**Published:** 2026-02-28

**Authors:** Tatiana M. Medvedeva, Lyudmila V. Vinogradova

**Affiliations:** Department of Molecular Neurobiology, Institute of Higher Nervous Activity and Neurophysiology, Russian Academy of Sciences, Butlerova Street 5A, 117485 Moscow, Russia; golova93tanya@gmail.com

**Keywords:** functional connectivity, spreading depolarization, seizure, electrophysiology, mutual information, phase synchronization, epilepsy, epileptogenesis

## Abstract

**Background:** Epilepsy is a network disorder, and network-based approaches to its diagnostics and therapies attract growing attention. Identification of prognostic markers of epileptogenesis and long-term risk for developing epilepsy after brain insults is an urgent, unresolved problem. We examined whether intracortical connectivity patterns reflect early epileptogenic changes in the cortex. **Methods:** We used the audiogenic kindling model, in which cortical epileptogenesis is initiated by repetition of reflex subcortically-driven seizures. Two measures of functional connectivity—mutual information and mean phase coherence—were applied to electrocorticographic recordings obtained from homotopical sites of parietal cortex during interictal and immediate postictal periods in awake rats. Interhemispheric connectivity and synchrony in non-kindled and slightly kindled rats were compared. Cortical spreading depolarization (SD), the first manifestation of growing cortical excitability in several models of epileptogenesis, was used as an electrographic marker of the earliest kindling stage. **Results:** In kindled animals, baseline levels of hemispheric connectivity and gamma band synchrony were significantly lower compared to seizure-naive rats. Before kindling, subcortical seizures elicited mild postictal depression of cortical gamma oscillations without changes in interhemispheric functional connectivity. Early in kindling, seizures produced wideband postictal depression of cortical activity and a striking drop in hemispheric connectivity. **Conclusions:** Primary network alterations during epileptogenesis involve hemispheric decoupling and reduced synchronization, both sustained (between seizures) and transient (postictal). Breakdown of long-range intracortical communication may reflect homeostatic plasticity and an active attempt to restrict epileptogenic reorganization of neural networks. We think that resting-state hemispheric hypocoupling could be an early marker of epileptogenesis. Seizure-induced SD contributes to the generation of postictal events.

## 1. Introduction

Identification of reliable biomarkers of epileptogenesis, a dynamic process leading to the transformation of the healthy brain to an epileptic one, remains an urgent task. Search for early predictors of epileptogenesis, allowing for selecting patients with high risk of epilepsy development after acute brain insults and starting preventive treatment, attracts growing attention. Network markers are a promising instrument for the purpose [1,2,3]. Pathophysiological mechanisms of epileptogenesis include widespread modification of inter-area functional interactions with recruitment of remote brain regions in the epileptic network. Pronounced network alterations have been reported during epileptogenesis in experimental animals [4,5] and patients with epilepsy [6,7,8]. However, very little is known about network changes at the early stage of epileptogenesis, which have no obvious clinical correlates.

Preclinical studies in different models of epileptogenesis have shown that spreading depolarization (SD), a wave of transient neuroglial depolarization [9], appears as the earliest cortical event at the initial subclinical stage of epileptogenesis when other signs of cortical hyperexcitability are subtle or absent [10,11]. In the model of human glioma, spontaneous episodes of cortical SD appear at the earliest stage of tumor-related epileptogenesis before the emergence of epileptiform activity in the cortex [10]. Cortical SD is reliably detected following brief mild ictal episodes at the initial stage of chemical and audiogenic kindling [11,12,13]. A close association of cortical SD with seizures, even subtle ones, has been reported in clinical [14,15] and preclinical [12,13,16] studies. Recently, intracranial stereotactic EEG showed the occurrence of cortical SD without obvious seizure activity in an epileptic patient [17]. However, the inability of conventional EEG to detect SD could lead to underestimation of its role in human epilepsy and make it difficult to study the seizure-SD association in the clinic.

Our previous study in the audiogenic kindling model has reported significant changes in interhemispheric functional connectivity during the middle and late periods of cortical epileptogenesis [18]. It has been shown that bilateralization of cortical seizures at the final kindling stage was accompanied by an increase in hemispheric connectivity, and the parietal cortex was very sensitive to the network changes [18]. Here, using the same experimental model, we focused on the connectivity changes in the parietal cortex at the early stage of kindling-related epileptogenesis. Well-known neuroanatomical substrates of audiogenic kindling and the use of only sensory (sound) stimulation for seizure initiation make the model especially valuable for studying network mechanisms of epileptogenesis. Primary epileptic focus (seizure-onset zone) of audiogenic seizures is located in the brainstem, and repetition of the subcortically-driven seizures leads to gradual recruitment of the cerebral cortex in the epileptic network [19,20]. The model mimics epileptogenic changes associated with hypothalamic hamartoma, in which seizures are generated by a subcortical lesion (hamartoma) and secondary spread to the cerebral cortex [21,22,23]. Kindling-like process is thought to underlie the development of cortical epileptiform activity in hamartoma-related epilepsy [21,22].

The mild version of audiogenic kindling used in our studies produces a slow stepwise progression of epileptogenic changes in the cortex. In the paradigm, a brief sound stimulation of susceptible rats induces a focal brainstem seizure behaviorally expressed as a brief episode of hyperkinetic seizure (unidirectional running). With repetition of subcortical seizures, the cortex is recruited in the epileptic network that manifests in appearance and gradual intensification of cortical seizures following brainstem seizure episodes [11,13,18]. At the early kindling stage before development of the kindled cortical seizures, unilateral SD is reliably detected in the cortex [11,13]. We suggested that interhemispheric functional connectivity starts to change early in kindling. To test the hypothesis, we compared long-term (interictal, resting state) and short-term (postictal) dynamics of hemispheric coupling before kindling and at the early kindling stage, using cortical SD as its electrographic marker. Time-related changes in functional connectivity were estimated by two measures—the mutual information (MI) function [24] and the index of phase synchronization (PS) [25] applied to LFP recordings from homotopic sites of the parietal cortex. We found that interhemispheric hypoconnectivity is a reliable manifestation of early epileptogenic changes in the cortex.

## 2. Materials and Methods

### 2.1. Animals

We used rats of the Wistar strain susceptible to audiogenic seizures (Stolbovaya breeding center, Moscow, Russia). Experiments were performed in adult males (350–450 g). Rats were housed in individual cages in a 12-h light–12-h dark lighting regime with free access to food and water. Experiments were conducted according to the ARRIVE guidelines and European Communities Council Directive (2010/63EU). The experimental protocol was approved by the Animal Care Committee of the Institute of Higher Nervous Activity and Neurophysiology (N1, 1 February 2022).

### 2.2. Surgery

Implantation of electrodes for recording wideband electrical activity of the cortex was performed two weeks before the onset of experiments. Recording electrodes (screws/glass pipette with inner carbon fiber) were implanted bilaterally in symmetrical points of the parietal cortex in rats treated with chloral hydrate (360 mg/kg, i.p.) and anesthetic lidocaine (locally). During the post-surgery period, rats received amoxicillin (0.3 mL, i.m.) and glucose solution (1 mL, s.c.). To avoid abdominal side effects, the feeding schedule was changed before the surgery (strict diet) and during the immediate post-surgery period (soft food). Coordinates of implantation were (mm from bregma): posterior 2.0 and lateral 3.0 [26]. Reference electrode (a stainless-steel screw) was positioned over the cerebellum. Electrodes were soldered to a pin connector and secured with acrylic cement.

### 2.3. Experimental Design

During experiments, local field potentials (LFP) were recorded in awake, freely behaving rats with simultaneous video-monitoring of their behavior. Animals were individually placed in an experimental chamber (60 × 40 × 40 cm) and connected to the recording cable. Electrical activity of the cortex (1 kHz sampling rate) was recorded with a four-channel, high-input impedance (1G) DC amplifier and A/D converter (E14-440, L-Card, Moscow, Russia) and stored on the computer for offline analysis. After a 5-min habituation and a 10-min baseline recording, a hyperkinetic seizure was induced by sound (13–85 kHz; 50–60 dB). Acoustic stimulation lasted until the seizure onset.

### 2.4. Audiogenic Kindling Procedure

Each rat received repeated sound stimulations once a day at 3–4-day intervals. Before kindling and at the early kindling stage, sound triggered only a single episode of hyperkinetic seizure (paroxysmal running). At the middle and final stages of kindling, the sound-induced hyperkinetic seizure was followed by limbic clonus (facial automatisms, ears/vibrissae clonus, head nodding) of growing duration and intensity. Data for the late kindling stages have been reported in our previous paper [18]. Here, we focused on connectivity dynamics during the early stage of kindling. We used cortical SD as an electrographic marker of growing cortical excitability. Two types of audiogenic seizures were analyzed: (1) non-kindled subcortical seizures (an episode of running) induced by the first and second sound stimulation; (2) slightly kindled subcortical seizures induced by repeated sound stimulation and accompanied by cortical SD. The seizure types were classified by two experienced observers, blinded to the groups. Based on LFP recordings and video data, the onset and termination of the ictal episode and SD were marked. SD was identified by a high-amplitude negative direct current potential shift. Latency of SD was determined by the time between the seizure termination and the SD onset.

### 2.5. Data Processing

The data segments were processed using Butterworth digital filters configured for high-pass (1 Hz cutoff) and band-stop (48–52 Hz) filtering. Recordings filtered with a lowpass (0–48 Hz) filter were used for the identification of SD. Subsequently, 300-s recordings were segmented into non-overlapping 10-s intervals, and the average spectral power for each interval and frequency band was calculated. The power spectrum was derived via fast Fourier transform (FFT) across five bands: delta (1–4 Hz), theta (4–8 Hz), alpha (8–12 Hz), beta (12–25 Hz), and gamma (25–50 Hz). Spectrograms were generated using an FFT block size of 2048 data points (equivalent to roughly 2 s) with a 90% overlap between consecutive windows.

### 2.6. Functional Connectivity Analysis

First, a blinded investigator selected 300-s epochs of local field potential (LFP) recordings free from artifacts. Data were obtained from homotopic sites in the parietal cortex under baseline and post-running conditions, both prior to kindling and in the early kindling stage. After selection, epochs were processed with a 1–100 Hz band-pass filter (Fourier transform, zero phase shift) for offline analysis. Postictal connectivity following running was calculated from LFP traces commencing right after the offset of a hyperkinetic seizure. Baseline connectivity was determined from 300 s of undisturbed background LFP, captured during a quiet period no less than 60 s before seizure induction. Each full epoch was further divided into non-overlapping 10-s intervals, and connectivity measures were computed for every interval. Finally, to assess coupling dynamics between hemispheres, we employed two computational tools: the mutual information function [24] and the phase synchronization index [27].

*Mutual information* (MI) quantifies the statistical dependency between two variables, X and Y. It shares the same units as Shannon’s information entropy and represents the amount of shared information contained within the variables. For our analysis, we implemented the nearest-neighbor estimation method introduced in [24]. In this framework, the joint state space is defined on an X-Y plane, where the X-axis corresponds to the original signal time series, and the Y-axis represents its quadrature component, derived via the Hilbert transform to form the analytic signal.

This estimation technique is based on the Kozachenko–Leonenko entropy estimator [28], which provides a robust computational approach to Shannon’s entropy. The calculation was performed using Formula (2):

(1) $$MI\left(x,y\right)=\psi \left(N\right)+\psi \left(1\right)-{\left\langle \psi \left(n_{x}\left(i\right)+1\right)+\psi \left(n_{y}\left(i\right)+1\right)\right\rangle }_{i=1,\ldots ,N},$$

In this formula, *N* represents the length of the time window in data points. For each i-th data point $\left(x_{i},y_{i}\right)$ on the joint (X,Y) plane, $n_{x}(i)$ and $n_{y}\left(i\right)$ denote the counts of neighbors whose distance from the point along the X or Y axis, respectively, is less than the distance to its overall nearest neighbor. The digamma function is represented by ψ(n). The estimator is asymptotically unbiased and delivers high precision for time windows where N>1000.

Mutual information is a well-established metric for detecting nonlinear signal similarities, particularly when the precise mechanisms of coupling are unknown or cannot be resolved with current experimental techniques. As an entropy-based information-theoretic measure, MI captures the total intensity of information exchange between the analyzed systems. This includes interactions across all frequency bands, mediated couplings, and nonlinear interactions. Comparative studies have demonstrated that MI offers considerable advantages over traditional correlation-based measures [29,30].

*Mean phase synchronization index*, as defined by Mormann et al. [25] (Equation (2)), quantitatively measures the simultaneity of phase changes between two signals.

(2) $$PS\left(x,y\right)=\frac{1}{N}\left|\sum_{n=1}^{N}exp\left(j\left(\varphi_{x}\left(t,n\right)-\varphi_{y}\left(t,n\right)\right)\right)\right|,$$

This index is calculated from the phase difference $\Delta \phi_{x,y}=\phi_{x}-\phi_{y}$, where $\phi_{x}$ and $\phi_{y}$ represent the instantaneous phases of the signals $\{x_{i}{\}}_{i=1}^{N}$ and $\{y_{i}{\}}_{i=1}^{N}$, respectively. The analytic signal was first constructed via the Hilbert transform. The instantaneous phase was then derived as the arc tangent in the complex plane. The presence of a stable rotational center in the complex plane for the analytic signal confirmed the validity of the phase estimation procedure.

Prior to analysis, signals were filtered into five standard frequency bands using a Fourier-based band-pass filter, consistent with the spectral analysis performed in this study. The band separation allowed for the calculation of a single, well-defined mean phase within each band. The estimated mean phase was found to be robust and accurate for nearly all-time segments in the delta and theta bands. Some estimation errors, however, were observed in the higher-frequency beta and gamma bands.

### 2.7. Statistical Analysis

Electrographic epileptic discharges duration is reported as mean ± standard error of the mean (S.E.M.). To evaluate temporal changes in connectivity measures, we computed, for each time point, the median and interquartile range across all recordings. The statistical null hypothesis stated that the median value of a measure at any given time point did not differ from its baseline median value. We aimed to reject this hypothesis at a per-comparison alpha level denoted as $\tilde{p}$, and the corrected significance level was:

(3) $$p={\tilde{p}}^{n}L$$

where *L* was the analysis interval (L=30 points in our case).

Subsequently, we applied a cluster-based permutation correction [31] to control multiple comparisons across time points. This method evaluates the significance of temporally adjacent points (clusters) rather than individual points. Within a defined time interval of length *L*, if *n* consecutive points each show a significant deviation from baseline at the uncorrected level $\tilde{p}$, and the cluster as a whole is assessed for significance. The corrected significance level *p* (Equation (3)) represents the probability that a cluster of at least size n would occur by chance under the null hypothesis, providing stronger control over false positives.

Within the analysis interval, we identified significant connectivity changes based on clusters of adjacent time points. A cluster was deemed significant if it contained at least three consecutive points (n=3) where measures individually differed from baseline p≤0.1. This cluster size corresponded to a corrected significance threshold of p≤0.003; larger clusters (n≥4) reached p<0.0005, while a two-point cluster (n=2) resulted in an insufficient corrected p-value>0.3. Significant intervals are marked with color in the figures. The Mann–Whitney U test was used to compare baseline connectivity across seizure types.

### 2.8. Software and Algorithms

The data analysis was conducted utilizing custom-developed scripts in Python 3.13, incorporating the following scientific libraries: Matplotlib 3.10.3 [32], NumPy 2.2.5 [33], and SciPy 1.15.3 [34].

## 3. Results

### 3.1. Electrographic Characteristics of Audiogenic Seizures at the Early Kindling Stage

In susceptible freely behaving rats, sound elicited a single hyperkinetic seizure—a brief (3–9 s) episode of self-sustained unidirectional running. In repeated tests, the direction of repeated running seizures remained consistent. After 9.0 ± 1.4 (range 6–15) repetitions, hyperkinetic seizures began to be followed by a unilateral SD detected in the cortex ipsilateral to the direction of running. In the parietal cortex, SD appeared 92.5 ± 4.7 s (*n* = 12) after termination of hyperkinetic seizures. Behavioral phenotype, intensity, and duration of kindled seizures with cortical SD and non-kindled seizures without SD were identical—6.7 ± 0.6 s vs. 6.8 ± 0.4 s, respectively (*n* = 12 per group). Hyperkinetic seizures terminated abruptly and were followed by postictal behavioral immobility. Its duration did not differ for seizures without SD (243 ± 27 s, *n* = 12) and with cortical SD (208 ± 21 s, *n* = 12, *p* = 0.3013, Mann–Whitney test).

**Figure 1:**
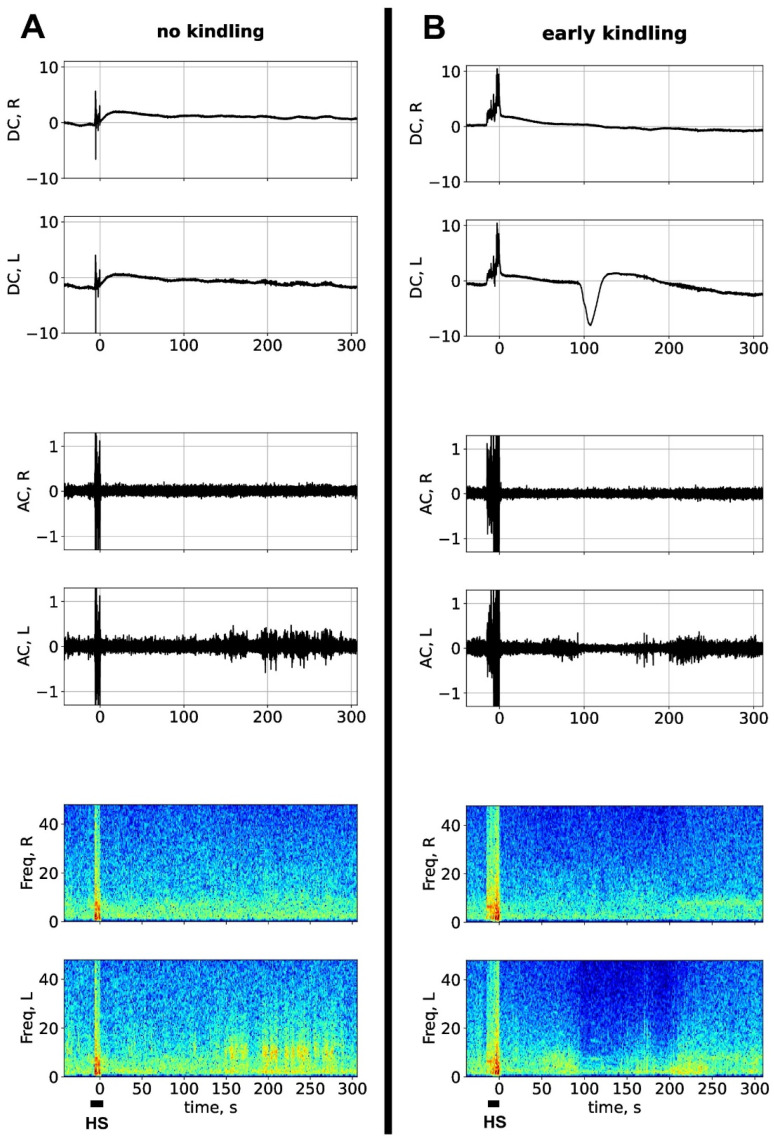
Electrographic pattern of sound-induced seizures before kindling and at the early kindling stage. (**A**,**B**) Representative DC (upper fragments) and AC recordings (middle fragments) obtained in homotopical sites of the parietal cortex of the left (L) and right (R) hemispheres of the same freely behaving rat before kindling (**A**) and at the early kindling stage (**B**). Respective spectrograms are shown below. Sound stimulation induced a brief episode of hyperkinetic seizure (HS) accompanied by high-amplitude artefacts (marked by a horizontal line). Early in kindling, spreading depolarization (large negative DC potential shift) appeared in the left parietal cortex 90 s after the end of the ictal episode (**B**). Ordinate is LFP amplitude in mV, abscissa is time in seconds (the “0” point marks the inset of postictal period).

Figure 1 shows representative DC/AC recordings of hyperkinetic seizures induced by the first sound stimulation in a seizure-naïve rat (A) and by the ninth acoustic stimulation in the same rat (B). The recordings were obtained in the homotopic regions of the parietal cortex of the two hemispheres during the peri-ictal period. High-amplitude artefacts mark the period of hyperkinetic seizure. After the ninth seizure (early kindling), a large negative DC potential shift (a signature of SD) appeared in the left cortex at about 90 s (Figure 1B). As seen in the spectrogram, non-kindled hyperkinetic seizure did not induce significant postictal changes in cortical activity (Figure 1A), but slightly kindled seizures associated with cortical SD produced transient depression of electrical activity, mainly in the left cortex affected by SD.

### 3.2. Postictal Depression of Cortical Activity at the Early Kindling Stage

**Figure 2:**
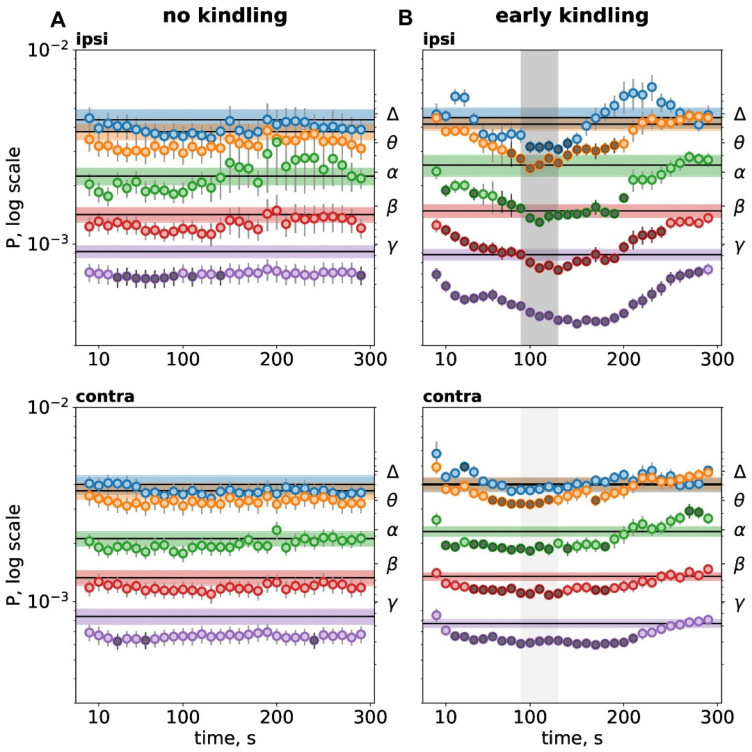
Spectral characteristics of postictal depression before kindling (**A**) and at the early kindling stage (**B**). Temporal dynamics of cortical oscillation power during the immediate postictal period (300 s). Graphs show mean power of delta (Δ, 1–4 Hz), theta (θ, 4–8 Hz), alpha (α, 8–12 Hz), beta (β, 12–25 Hz), and gamma (γ, 25–50 Hz) oscillations (the right Y-axis) in the parietal cortex ipsilateral (ipsi) and contralateral (contra) to run direction/SD (n = 12). Within each band, horizontal lines with shadows show baseline activity power (mean ± SEM). Circles mark power for each 10-s interval of the postictal period, and dark circles indicate intervals significantly different from the baseline level (*p* < 0.05, one-way ANOVA for repeated measures). Zero point corresponds to seizure termination and the onset of the postictal period. Gray vertical areas in B mark duration of the depolarization phase of SD.

Spectral analysis showed that non-kindled subcortical seizures produced mild postictal depression of high-frequency gamma oscillations without changes in other frequency bands (Figure 2A). The gamma depression lasted longer in the cortex of one hemisphere (90 s in total) than in another (20 s). The asymmetry of the gamma depression corresponded to motor asymmetry of hyperkinetic seizures—depression was longer in the cortex ipsilateral to the direction of running and homolateral to the brainstem seizure focus. This was the cortex where SD and kindled seizure appeared first during kindling.

At the early kindling stage, when unilateral SD appeared in the cortex, the postictal depression became strong and wideband. Fast (beta-gamma) cortical oscillations showed longer (230–280 s) and stronger depression compared to slow cortical activity. In the cortex affected by SD, beta-gamma depression started long before SD arrival at the recording site and subsided after SD termination. The power of the slowest delta activity was reduced only during SD and only in the cortex affected by SD. In the contralateral cortex unaffected by SD, cortical oscillations in all frequency bands except delta were also depressed, though milder than in the SD-affected cortex.

### 3.3. Homotopic Functional Connectivity During Postictal and Interictal (Baseline) Periods Before Kindling and at the Early Kindling Stage

#### 3.3.1. Mutual Information

**Figure 3:**
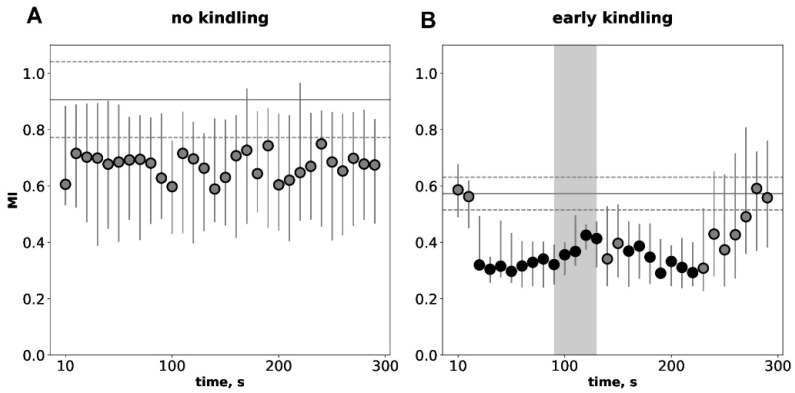
Temporal dynamics of interhemispheric functional connectivity quantified by the mutual information (MI) analysis during the postictal period before kindling (**A**) and at the early kindling stage (**B**). The connectivity between homotopic sites of the parietal during the immediate 300-s postictal period (circles) as compared to the pre-seizure baseline period (horizontal lines). Ordinate is the MI level. Gray vertical area in B marks duration of the depolarization phase of SD. Other abbreviations are as in Figure 2.

Temporal dynamics of mutual information (MI) values during a 300-s immediate postictal period compared to baseline preictal levels for non-kindled and slightly kindled seizures are shown in Figure 3. Before kindling, hyperkinetic seizures did not change the baseline level of MI (Figure 3A). At the early kindling stage, audiogenic seizures produced an abrupt twofold decrease in connectivity strength for several minutes (Figure 3B). The MI drop had a two-wave pattern with the first wave (30–140 s) starting before SD arrival to the recording site and the second wave (170–230 s) developing after termination of cortical SD. Baseline MI level was significantly lower in slightly kindled animals compared to seizure-naïve ones (*p* < 0.05, *p* = 0.03791; Mann–Whitney test, Figure 3). Thus, MI analysis shows that the early stage of epileptogenesis is associated with a pronounced decrease in interhemispheric functional connectivity both during interictal and postictal periods.

#### 3.3.2. Phase Synchronization

**Figure 4:**
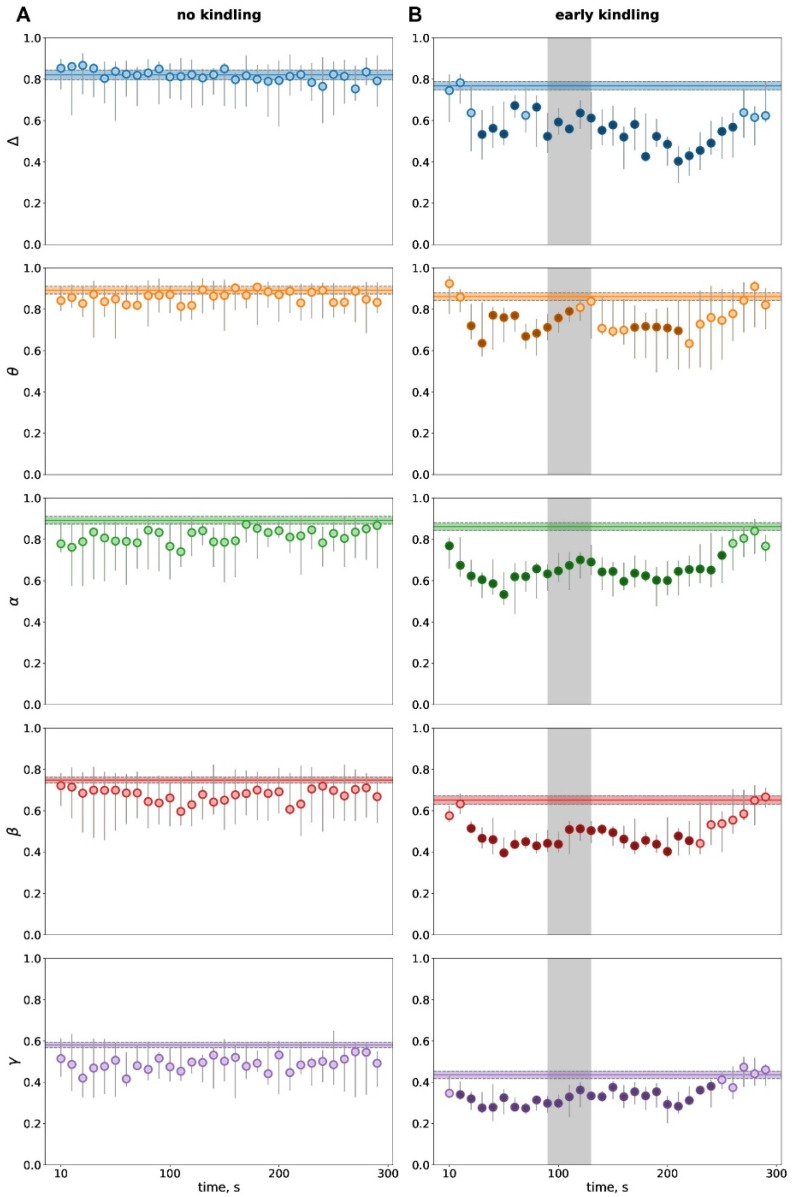
Temporal dynamics of interhemispheric phase synchronization (PS) during the postictal period before kindling (**A**) and at the early kindling stage (**B**). Phase synchronization between homotopic sites of the parietal cortex was quantified for five frequency bands (delta, theta, alpha, beta, gamma) during 300-s postictal periods as compared to pre-seizure baselines. Ordinate is the PS level. Gray vertical areas in B mark duration of the depolarization phase of SD. Other abbreviations are as in Figure 2 and Figure 3.

Interhemispheric phase synchronization (PS) showed no changes following non-kindled subcortically driven seizures (Figure 4A). With recruitment of the cortex in the epileptic network and appearance of cortical SD, hyperkinetic seizures started to induce transient wideband loss of interhemispheric synchronization during the postictal period (Figure 4B). The hemispheric desynchronization started very soon after seizure termination—immediately in the alpha band and a bit later (since 20–40 s) in other frequency bands. The drop in interhemispheric synchronization lasted till 220–270 s.

Comparison of baseline levels of interhemispheric synchronization in seizure-naïve and slightly kindled animals showed no significant difference in the delta (*p* = 0.178), theta (*p* = 0.307), and alpha (*p* = 0.307) frequency bands but a tendency toward lowering in the beta band (*p* = 0.066) and a significant reduction in the gamma band (*p* = 0.004) at the early kindling stage.

## 4. Discussion

### 4.1. Reduced Resting-State Hemispheric Connectivity at the Early Stage of Epileptogenesis

Our previous study in the audiogenic kindling model has shown that resting-state hemispheric connectivity significantly changes during late-stage kindling associated with the development of cortical seizures [18]. The present findings demonstrate a very early onset of the network reorganization, long before the epileptic activation of the cortex, and baseline interhemispheric connectivity is reduced compared to its pre-kindling level. Given the data, we can trace the evolution of the resting-state connectivity over the duration of kindling-related epileptogenesis. Baseline MI level is reduced early in kindling (present study) and remains reduced later [18], which indicates persistent functional decoupling of the two cortices during epileptogenesis. The result is in line with clinical data from resting-state fMRI studies showing reduced functional connectivity between homologous temporal lobes of the two hemispheres in patients with temporal lobe epilepsy compared to healthy controls [6,35,36].

Our analysis of phase synchronization has shown that the hemispheric synchrony in the gamma frequency band is the most sensitive marker of epileptogenesis compared to other bands. As shown in our previous study, baseline gamma connectivity between the hemispheres increases at the final stages of kindling, when cortical seizures become bilateral, which suggests a role of the gamma hypercoupling in facilitated spread of epileptic activity between the cortex of the two hemispheres [18]. The present study demonstrates reduced gamma synchronization of the hemispheres at the early kindling stage. Therefore, the data from our present and previous studies show that kindling progression is associated with early hemispheric gamma-band hyposynchrony followed by gamma hypersynchronization. Similar alterations of resting-state fMRI connectivity with transformation from hyposynchrony to hypersynchrony have been described during epileptogenesis in post-status animal models [5]. The interhemispheric synchronization in the gamma frequency band may be a very sensitive marker of epileptogenic changes in the cortex. Given that cortical gamma activity plays a critical role in cognition, the alterations of gamma synchrony may underlie cognitive dysfunction in epileptic patients.

Alterations in baseline hemispheric connectivity reflect long-term plasticity of neural networks that can either promote or prevent expansion of the epileptic network. We suggest an adaptive role of the early functional disconnection of the hemispheres. The sustained decrease in hemispheric communication early in epileptogenesis is likely to represent a homeostatic network mechanism preventing or hindering seizure spread within the brain. This is in line with clinical studies that reported reduced LFP synchrony between the seizure-onset zone and surrounding brain regions [37]. The authors suggested that the hyposynchrony produces functional isolation of the epileptic focus from other brain regions.

### 4.2. Postictal Dynamics of Interhemispheric Connectivity at the Early Stage of Epileptogenesis

At the early kindling stage, in addition to the sustained interhemispheric hypoconnectivity, seizures began to be followed by a transient postictal drop in hemispheric coupling. In non-kindled animals, brainstem seizures do not change interhemispheric connectivity during the postictal period. This indicates that locomotor excitation and stress associated with the seizures do not affect functional interactions between hemispheres. In slightly kindled rats, similar seizures are followed by a twofold drop in hemispheric connectivity (both MI and PS) for several minutes. Given our previous findings [18], we observe dynamic modification of postictal connectivity patterns during epileptogenesis. Postictal MI drop appears at the early stage of kindling, disappears at the middle stage (no changes after focal cortical seizures), and is replaced by postictal hyperconnectivity at the final kindling stage (increased MI after bilateral cortical seizures). Postictal PS levels show similar dynamics during epileptogenesis—a wideband drop early in kindling, which becomes milder or disappears later and progresses to delta hypercoupling at the final stage of kindling [18]. Thus, postictal changes in interhemispheric connectivity evolve from hypo- to hyperconnectivity during epileptogenesis. Postictal alterations in brain activity are thought to reflect reversible seizure-induced changes in network activity that may include adaptive mechanisms of short-term plasticity directed at restoration of brain function. We suggest that the homeostatic mechanisms are activated early during kindling in a relatively normal brain but become exhausted with recurrent seizures and finally convert to maladaptive changes promoting seizure propagation in the hyperexcitable brain. The post-seizure breakdown of hemispheric connectivity and disruption of functional integrity of brain networks may underlie reversible deficits in perceptual, executive, and cognitive function during the postictal period.

### 4.3. Postictal Depression of Cortical Activity Before Kindling and at the Early Kindling Stage

Transient neurological deficit and EEG depression are well-recognized postictal events in epileptic patients. In our study, subcortically driven seizures are followed by mild depression of cortical gamma oscillations before kindling and by strong, wideband depression early in kindling. For both non-kindled and kindled seizures, maximal postictal depression was found in the high-frequency gamma band. Cortical gamma activity plays a crucial role in higher brain function, including perception and arousal. Suppression of gamma oscillations in the cortex is associated with a decrease in arousal [38]. As shown in the present study, audiogenic seizures were always followed by postictal behavioral immobility. Behavioral arrest in rodents is thought to reflect dysfunction of brainstem mechanisms controlling movement initiation [39]. Maintenance of gamma activity in the cortex is related to cortical networks and ascending drive from the pedunculopontine nucleus [38,40]. The brainstem nucleus is a part of the reticular activating system and mesencephalic locomotor regions, which play a role in the regulation of arousal state and locomotion [19,38]. Deficient cortical gamma activity during the postictal period found in our study may reflect abnormalities in the rhythm-generating networks. An important role of the networks in control of arousal and movement initiation may explain the association of gamma depression with the decrement in arousal levels and behavioral arrest during the postictal period.

### 4.4. Cortical SD Is an Electrographic Marker of Early Epileptogenic Changes and a Contributing Factor in Postictal Alterations of Cortical Activity

Cortical spreading depolarization (SD) develops in response to different brain insults, including epileptic seizures [9,12,14,16,41]. Co-occurrence of cortical SD and seizures has been reported in clinical [14,15] and preclinical [12,13,16] studies. It is suggested that SD triggered by focal cortical seizures can act as an endogenous anti-seizure mechanism [16]. In several animal models, cortical SD appears at the initial subclinical stage of epileptogenesis before the emergence of pronounced epileptiform activity in the cortex [10,11]. Given that standard scalp electroencephalography cannot detect SD, its occurrence in the human epileptic brain may be underestimated. In a recent study using intracranial stereotactic electroencephalography, cortical SD was detected in an epileptic patient during presurgical evaluation [17].

In audiogenic kindling, cortical SD can be triggered either by ascending drive from the brainstem seizure focus or by mild epileptic activation of the cortex during brainstem seizures. In either case, triggering cortical SD indicates a hyperexcitable state of the cortex. In audiogenic kindling, cortical SD appears with a large time lag (90–100 s) after termination of a brainstem-driven seizure. It looks as if SD arises in a remote circumscribed region of the cortex and slowly propagates to the parietal cortex (velocity of SD propagation over the cortex is 3–6 mm/min). Unilateral pattern of cortical SD confirms the focal triggering of cortical SD and suggests non-symmetric growing excitability of the cortex of the two hemispheres during audiogenic kindling.

Postictal changes in cortical activity at the early kindling stage seem to represent interference of the depressive effects of seizures and seizure-induced SD. Pattern of postictal depression after slightly kindled seizures—strong silencing fast (beta-gamma) activity and mild suppression of slow cortical oscillations—is similar to that induced by cortical SD in healthy awake rats [42]. A role of SD in postictal depression is also supported by the longer and stronger suppression of spontaneous electrical activity in the cortex affected by SD. On the other hand, the rapid onset of postictal depression and involvement of the cortex unaffected by SD also indicates a contribution of ictal activity. Both seizure and seizure-induced SD are likely to contribute to postictal hemispheric disconnection. It has a two-wave pattern. The first wave develops before SD arrival at the recording point and seems to result from ictal excitation of the cortex. Timing of the second wave corresponds well to a network signature of unilateral cortical SD—transient (100 s) interhemispheric decoupling which starts after termination of SD [43,44].

## 5. Conclusions

Traditionally, epilepsy is considered a disorder of excessive synchronization and hyperconnectivity. Our findings have revealed that the earliest phase of epileptogenesis is associated with reduced synchronization and breakdown of normal long-range communication (decoupling of the hemispheres) during interictal and postictal periods. The initial reduction in hemispheric communication may reflect homeostatic long- and short-term plastic alterations within brain networks and an active attempt of the brain to restrict the epileptogenic process and to isolate functionally the cortical regions experiencing seizure or subcortical drive at the initial stage of epileptogenesis. Enhanced synchronization and hypercoupling might be local or develop later during the epileptogenic process. Our findings suggest that seizure-induced SD (spreading depolarization) contributes to hemispheric decoupling and depression of cortical activity during the postictal period. This indicates that postictal changes reflect not just neural exhaustion but result from active events triggered by seizures. We think that hemispheric hypoconnectivity (resting-state and postictal) may be used as an early marker of acquired epilepsy. Identification of its markers, which allows for predicting the long-term risk for development of epilepsy after acute brain insults (stroke, trauma), is an urgent unresolved problem [3,45]. We believe that our findings may offer clues to its resolution.

## Data Availability

The datasets used and analyzed during the current study are available from the corresponding author upon reasonable request.
